# Sex differences in serum levels of 5α-androstane-3β, 17β-diol, and androstenediol in the young adults: A liquid chromatography–tandem mass spectrometry study

**DOI:** 10.1371/journal.pone.0261440

**Published:** 2021-12-15

**Authors:** Haruka Tanabe, Hitoshi Mutai, Daimei Sasayama, Hidehiko Sasamoto, Yoshimichi Miyashiro, Nobuhiro Sugiyama, Shinsuke Washizuka

**Affiliations:** 1 Department of Medical Sciences, Graduate School of Medicine, Science and Technology, Shinshu University, Matsumoto, Nagano, Japan; 2 Department of Psychiatry, Shinshu University School of Medicine, Matsumoto, Nagano, Japan; 3 Division of Health Sciences, Department of Medical Sciences, Graduate School of Medicine, Shinshu University, Matsumoto, Nagano, Japan; 4 Mental Health Clinic for Children, Shinshu University Hospital, Matsumoto, Nagano, Japan; 5 Child and Adolescent Developmental Psychiatry, Shinshu University School of Medicine, Matsumoto, Nagano, Japan; 6 ASUKA Pharma Medical Co., Ltd. Shonan Health Innovation Park, Fujisawa, Kanagawa, Japan; 7 Department of Applied Occupational Therapy, Shinshu University School of Health Sciences, Matsumoto, Nagano, Japan; Kumamoto University Faculty of Life Sciences School of Medicine: Kumamoto Daigaku Daigakuin Seimei Kagaku Kenkyubu Igakubu, JAPAN

## Abstract

Animal experiments have consistently shown that estrogen receptor β (ERβ)-selective ligands have antidepressant and anxiolytic effects. In humans, endogenous ligands for ERβ include 5α-androstane-3β, 17β-diol (3βAdiol) and androstenediol (Δ5-diol). We determined, for the first time, the exact serum levels of 3βAdiol and Δ5-diol in young healthy volunteers using liquid chromatography–tandem mass spectrometry (LC–MS/MS). We investigated the effect of the menstrual cycle on the levels of these steroids in women; then, we performed a gender comparison. Blood samples were collected from 48 subjects: 23 women (mean age = 28.4±7.8 years) and 25 men (mean age = 31.4±7.8 years). We collected the blood samples of women at three time-points in the menstrual cycle: the early follicular phase, ovulatory or mid-cycle phase, and mid-luteal phase. A total of 92 blood samples were analyzed using LC–MS/MS. The levels of two well-studied steroids, namely dehydroepiandrosterone (DHEA) and 17β-estradiol (E2), were simultaneously measured. Depression rating scale (Hamilton Rating Scale for Depression, Beck Depression Inventory-II and Quick Inventory of Depressive Symptomatology) scores were also recorded at the time of blood sampling. Significant differences in the levels of 3βAdiol and E2 and in the depression rating scale scores were observed over the duration of the menstrual cycle of the women. The levels of 3βAdiol and Δ5-diol were significantly lower in women than in men. E2 levels were higher in women than in men, and DHEA levels did not differ significantly between men and women. Further, women had higher scores than men on the Hamilton Rating Scale for Depression. Sex differences in depressive symptoms can be explained by 3βAdiol and Δ5-diol levels, and the effect of the menstrual cycle on mood can be explained by 3βAdiol and E2 levels, not by Δ5-diol level.

## Introduction

The physiological functions of the novel estrogenic steroids 5α-androstane-3β, 17β-diol (3βAdiol) [1, 2] and androstenediol (Δ5-diol) [3] have been clarified in recent studies. Two types of estrogen receptors (ERs), namely ERα and ERβ, play a central role in ER signaling [4]. Selective stimulation of ERβ, not ERα, produces antidepressant effects [5–7]. Of the biosynthesized steroids, 3βAdiol and Δ5-diol have been identified as endogenous ERβ-selective agonists [4, 8]. Results of animal experiments strongly suggest that 3βAdiol [9–11] and Δ5-diol [3] are antidepressants. However, studies on these steroids in human subjects are few.

In our previous study [8], we measured the serum levels of 3βAdiol and Δ5-diol in subjects over 60 years of age and found that men have about five times higher 3βAdiol levels and about two times higher Δ5-diol levels than women. In this study, younger healthy men and women aged 20 to 45 were included. Since this study included pre-menstrual women of reproductive age, we needed to investigate the effects of the menstrual cycle on 3βAdiol and Δ5-diol levels, which were then compared to those of age-matched men.

It is challenging to measure low steroid levels in blood samples, as the accuracy, sensitivity, and specificity of assays are always concerns [12]. Therefore, we measured steroid levels using liquid chromatography–tandem mass spectrometry (LC–MS/MS), which is currently regarded as the most widely accepted and reliable assay. Dehydroepiandrosterone (DHEA; a precursor of 3βAdiol and Δ5-diol) [13] and 17β-estradiol (E2) [14, 15] were simultaneously measured as positive controls since their serum levels has been studied in detail in both sexes and in subjects of all ages. Furthermore, at each blood draw, an experienced psychiatrist determined the scores of each subject on depression rating scales. This study presents the first comprehensive data on the natural ERβ ligands, 3βAdiol and Δ5-diol, and provides deeper insight into the association between the serum levels of these steroids and depressive symptoms in young healthy adults.

## Materials and methods

### Study participants

This study was approved by the Ethics Committee of Shinshu University School of Medicine, Japan (study approval number: 3920). Between 2018 and 2021, volunteers were recruited locally using flyers approved by the committee. The inclusion criteria are as follows: (a) good health, age of 20–45 years, and ability to provide informed consent and (b) (for women) confirmed start and end dates of the previous three menstrual cycles, stable menstrual cycle (i.e., menstrual cycle length of 25–38 days with a variation of ±6 days), and menstrual period length of 3–7 days. The exclusion criteria are as follows: (a) severe general medical condition, (b) dementing illness or mild cognitive impairment, (c) daily use of drugs known to alter sex hormone balance (e.g., contraceptives and anti-estrogen drugs), and (d) history of orchiectomy or ovariectomy. A total of 48 subjects (mean age ± standard deviation [SD] = 30±7.9 years) consisting of 23 women (mean age ± SD = 28.4±7.8 years) and 25 men (mean age ± SD = 31.4±7.8 years) were enrolled. After describing the study, written informed consent was obtained from all study participants. Height and weight were measured, and body mass index (BMI) was calculated. The mean ± SD of BMI was 23.4±2.5 kg/m^2^ in men and 21.0±2.3 kg/m^2^ in women. The date of onset of the next menstrual cycle in women was predicted based on menstrual records.

### Blood sample collection

The blood samples of women were collected at three time-points in their menstrual cycle. The first sample was collected in the early follicular phase (EFP; days 1–5). During their initial visit, the women were asked to monitor their morning urine samples 3–5 days before their expected day of ovulation using luteinizing hormone (LH) surge detection kits (CheckOne LH II; ARAX, Nagoya, Japan) provided at the time of study consent. The second blood sample was collected in the periovulatory or midcycle phase (i.e., Midcycle) within 48 hours after LH surge detection. The third blood sample was collected in the mid-luteal phase (MLP) 7–10 days after ovulation. We confirmed that all third visits were before the start of the next menstrual period. In contrast, the blood samples of men were collected only once. A total of 92 blood samples were collected at the same time of day (around 10:00 AM) to avoid circadian variations in steroid levels. At the time of blood collection, the subjects were in the same position (i.e., seated on a chair with arms placed on arm rests) and in a quiet and relaxed state. The blood samples were centrifuged, and the sera were stored at −80°C pending steroid level measurements.

### Steroid level measurements

LC–MS/MS was used for the quantification of the serum levels of 3βAdiol, Δ5-diol, DHEA, and E2, with few modifications to the method described in our previous study [8].

#### Extraction and purification

DHEA-^13^C_3_, Δ5-diol-d_4_, 3βAdiol-d_3_, and E2-^13^C_4_ were added to the serum samples as internal standards. The steroids were extracted using methyl *tert*-butyl ether. After the organic layer was evaporated to dryness, the extract was dissolved in 0.5 mL of methanol and diluted with 1 mL of distilled water. The sample was applied to an OASIS MAX cartridge, which had been successively conditioned with 3 mL of methanol and 3 mL of distilled water. After the cartridge was washed with 1 mL of distilled water, 1 mL of methanol/distilled water/acetic acid (45:55:1, v/v/v), and 1 mL of 1% pyridine solution, the steroids were eluted with 1 mL of methanol/pyridine (100:1, v/v).

#### Derivatization and application to LC–MS/MS

After evaporation, the residue was reacted with 50 μL of a mixed solution (80 mg of 2-methyl-6-nitrobenzoic anhydride, 20 mg of 4-dimethylaminopyridine, and 40 mg of picolinic acid in 1 mL of acetonitrile) and 10 μL of triethylamine at room temperature for 30 min. After the reaction, the sample was dissolved in 0.5 mL of ethyl acetate/hexane/acetic acid (15:35:1, v/v/v), and the mixture was applied to an InertSep SI cartridge, which had been successively conditioned with 3 mL of acetone and 3 mL of hexane. The cartridge was washed with 1 mL of hexane and 2 mL of ethyl acetate/hexane (3:7, v/v), and the steroids were eluted with 2.5 mL of acetone/hexane (7:3, v/v). After evaporation, the residue was dissolved in 0.1 mL of acetonitrile/distilled water (2:3, v/v), and the solution was subjected to LC–MS/MS.

For the quantification of steroid levels, the transitions *m/z* 394.3→175.1, 397.4→178.4, 501.3→255.4, 505.4→259.3, 503.3→257.1, 506.3→260.1, 483.2→264.0, and 487.2→268.0 were selected for DHEA, DHEA-^13^C_3_, Δ5A-diol, Δ5A-diol-d_4_, 3βAdiol, 3βAdiol-d_3_, E2, and E2-^13^C_4_, respectively. The limits of quantification of DHEA, Δ5-diol, 3βAdiol, and E2 were 10 pg/mL, 5 pg/mL, 2.5 pg/mL, and 5 pg/mL, respectively.

### Assessment of mood

The mood of subjects was assessed when they visited the hospital for blood sampling. The scores of the subjects on the three depression assessment scales (Hamilton Rating Scale for Depression 21 items [HAM-D] [16], Beck Depression Inventory-II [BDI-II] [17, 18], and Quick Inventory of Depressive Symptomatology-Japanese version [QIDS-J] [19]) were recorded. A skilled psychiatrist (H.T.) conducted a one-on-one interview with each subject and determined the HAM-D score of each patient. BDI-II and QIDS-J are commonly used as self-rating inventories.

### Statistical analysis

The data obtained were assessed for normality using Shapiro–Wilk test; thereafter, the appropriate statistical tests for analysis were performed. Differences in serum steroid levels and scores on depression inventories at the three time-points in the menstrual cycle (i.e., the EFP, Midcycle, and MLP) were assessed using the Friedman tests. Post hoc analysis was conducted using Wilcoxon signed-rank test, with application of the Bonferroni correction. One female subject provided data only in the EFP and not in the Midcycle and MLP. The Friedman test was performed after the exclusion of this sample. To evaluate sex differences (men versus women in the EFP, Midcycle, or MLP) in serum steroid levels and scores on depression inventories, group mean comparisons were performed using Kruskal–Wallis test. If the results of Kruskal–Wallis test were significant, differences between pairs of men and women (in one of EFP, Midcycle, or MLP) were evaluated through multiple comparisons using Steel–Dwass test. Associations between serum steroid level and score on depression inventories were assessed using Spearman’s correlation coefficients.

The serum steroid levels obtained in this study were compared to those reported in a previously published study on geriatric subjects (as the sample collection protocols in the studies are identical) [8]. The comparisons between the young and the old were assessed using Mann–Whitney test for men and Kruskal–Wallis test followed by Steel–Dwass test for women.

A value of P < 0.05 was considered statistically significant. All analyses were performed using JMP version 13.2.0 (SAS Institute Japan) and statistical package for the social sciences (SPSS) version 27 (IBM Corp., Armonk, NY).

## Results

### Overall view

**Fig 1:**
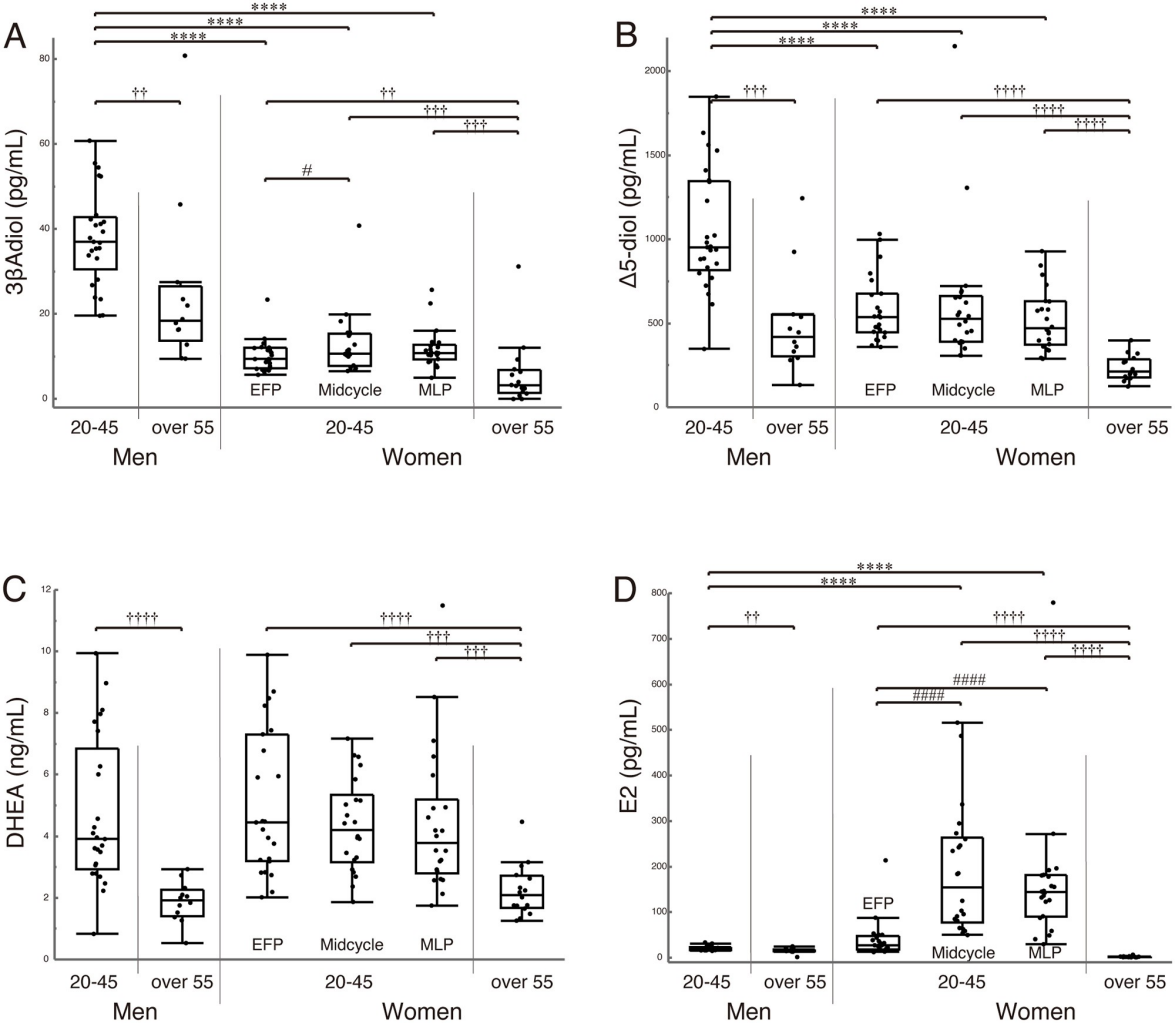
Serum levels of steroids (overall view). Serum levels of 3βAdiol (A), Δ5-diol (B), DHEA (C), and E2 (D) in young men aged 20–45, elderly men over 55 years old, young women aged 20–45 at three time-points in their menstrual cycle (EFP, Midcycle, and MLP), and elderly women over 55 years old. Data on elderly men and women are from our previous publication [8]. Median serum levels of each steroid are indicated in each box using horizontal bars. The vertical bars indicate the range, and the horizontal boundaries of each box represent the first and third quartiles. **** p<0.0001 as compared between young men and women; # p<0.05, #### p<0.0001 as compared during the menstrual cycle (at EFP, Midcycle, and MLP); †† p<0.01, ††† p<0.001, †††† p<0.0001 as compared between young and elderly subjects. *Abbreviations*: *3βAdiol*, 5α-androstane-3β,17β-diol; *Δ5-diol*, androstenediol; *DHEA*, dehydroepiandrosterone; *E2*, 17β-estradiol; *EFP*, early follicular phase; *Midcycle*, mid-cycle phase; *MLP*, mid-luteal phase.

Box-and-whisker plots of the measured serum levels of each steroid are shown in Fig 1. The data on elderly men and women are from our previous publication [8].

### Steroid hormone levels in women during the menstrual cycle (Table 1)

The mean 3βAdiol level changed significantly over the three time-points in the menstrual cycle (p = 0.0062), with Midcycle levels 20% higher than EFP levels at 12.80±7.31 pg/mL and 10.13±3.78 pg/mL, respectively (p = 0.031). There were no significant changes in serum Δ5-diol level over the time-points in the menstrual cycle (p = 0.142). As expected, serum E2 levels changed dynamically, peaking in the Midcycle and falling to minimal levels in the EFP (p<0.0001). In contrast, serum DHEA levels were unchanged throughout the cycle (p = 0.422).

**Table 1:** Serum steroid levels at three time-points in the menstrual cycle.

	Women			*Friedman test*	*Wilcoxon signed-rank test (with Bonferroni correction)*
	EFP (n = 22)	Midcycle (n = 22)	MLP (n = 22)		
3βAdiol (pg/mL)	10.13 (3.78)	12.80 (7.31)	11.85 (4.60)	**p = 0.0062**	**EFP vs Midcycle (p = 0.031)**
Δ5-diol (ng/mL)	0.58 (0.20)	0.62 (0.40)	0.52 (0.18)	p = 0.142	
DHEA (ng/mL)	5.08 (2.40)	4.36 (1.49)	4.43 (2.33)	p = 0.422	
E2 (pg/mL)	37.97 (41.44)	187.91 (135.78)	163.86 (149.25)	**p<0.0001**	**EFP vs Midcycle (p<0.0001)**
					**EFP vs MLP (p<0.0001)**

The differences at the three time-points in the menstrual cycle (EFP, Midcycle, and MLP) were assessed using the Friedman test. Post hoc analysis was conducted using Wilcoxon signed-rank test, with application of the the Bonferroni correction. The values are expressed as mean (SD).

*Abbreviations*: *3βAdiol*, 5α-androstane-3β,17β-diol; *Δ5-diol*, androstenediol; *DHEA*, dehydroepiandrosterone; *E2*, 17β-estradiol; *EFP*, early follicular phase; *Midcycle*, mid-cycle phase; *MLP*, mid-luteal phase

### Mood of women at three time-points in the menstrual cycle (Table 2)

There were significant differences in the mean scores for HAM-D, BDI-II, and QIDS-J over the three time-points in the menstrual cycle (p = 0.0066, 0.0011, and 0.017, respectively). The mean score on HAM-D was significantly higher in the EFP (3.2±2.6) than in the Midcycle (1.4±1.7, p = 0.012) and in the MLP (1.9±2.5, p = 0.045). The V-shaped pattern of scores was also observed with BDI-II and QIDS-J. The scores for each subitem of HAM-D, BDI-II, and QIDS-J are shown in S1 Table.

**Table 2:** Scores on depression rating scales during the menstrual cycle.

	Women			*Friedman test*	*Wilcoxon signed-rank test (with Bonferroni correction)*
	EFP (n = 22)	Midcycle (n = 22)	MLP (n = 22)		
HAM-D	3.2 (2.6)	1.4 (1.7)	1.9 (2.5)	**p = 0.0066**	**EFP vs Midcycle (p = 0.012)**
					**EFP vs MLP (p = 0.045)**
BDI-II	6.2 (6.1)	3.0 (3.7)	3.4 (3.8)	**p = 0.0011**	**EFP vs Midcycle(p = 0.0067)**
QIDS-J	3.6 (3.3)	2.3 (2.6)	2.3 (2.2)	**p = 0.017**	

The differences at the three time-points in the menstrual cycle (EFP, Midcycle, and MLP) were assessed using the Friedman test. Post hoc analysis was conducted using Wilcoxon signed-rank test, with application of the Bonferroni correction. The values are expressed as mean (SD).

*Abbreviations*: *BDI-II*, Beck Depression Inventory-II; *EFP*, early follicular phase; *HAM-D*, Hamilton Rating Scale for Depression 21 items; *Midcycle*, mid-cycle phase; *MLP*, mid-luteal phase; *QIDS-J*, Quick Inventory of Depressive Symptomatology-Japanese version

### Sex differences in serum steroid levels (Table 3)

Statistically significant differences in the serum levels of 3βAdiol, Δ5-diol, and E2 were observed between the sexes. Men had higher serum levels of 3βAdiol (mean ± SD = 38.02±11.13 pg/mL) and Δ5-diol (mean ± SD = 1.04±0.36 ng/mL) than women in all three time-points in the menstrual cycle. In contrast, men had lower serum levels of E2 (mean ± SD = 21.00±4.71 pg/mL) than women in the Midcycle (mean ± SD = 187.91±135.78 pg/mL) and women in the MLP (mean ± SD = 163.86±149.25 pg/mL). However, there were no statistically significant differences in serum E2 level between men (mean ± SD = 21.00±4.71 pg/mL) and women in the EFP (mean ± SD = 40.14±41.81 pg/mL) (p = 0.1101). As expected, significant differences in serum DHEA level were not observed between the sexes (p = 0.777).

**Table 3:** Sex differences in serum steroid levels.

	Men (n = 25)	Women			*Kruskal–Wallis test*	*Steel–Dwass test*
		EFP (n = 23)	Midcycle (n = 22)	MLP (n = 22)		
3βAdiol (pg/mL)	38.02 (11.13)	10.22 (3.72)	12.80 (7.31)	11.85 (4.60)	**p<0.0001**	**Men vs EFP (p<0.0001)**
						**Men vs Midcycle (p<0.0001)**
						**Men vs MLP (p<0.0001)**
Δ5-diol (ng/mL)	1.04 (0.36)	0.58 (0.20)	0.62 (0.40)	0.52 (0.18)	**p<0.0001**	**Men vs EFP (p<0.0001)**
						**Men vs Midcycle (p<0.0001)**
						**Men vs MLP (p<0.0001)**
DHEA (ng/mL)	4.70 (2.40)	5.06 (2.35)	4.36 (1.49)	4.43 (2.33)	p = 0.777	
E2 (pg/mL)	21.00 (4.71)	40.14 (41.81)	187.91 (135.78)	163.86 (149.25)	**p<0.0001**	**Men vs Midcycle (p<0.0001)**
						**Men vs MLP (p<0.0001)**

Serum levels of four steroids (3βAdiol, Δ5-diol, DHEA, and E2) were compared between men and women in the EFP, Midcycle, and MLP. Four group mean comparisons were performed using Kruskal–Wallis test. If the results of Kruskal-Wallis test were significant, differences between pairs of men and women (in one of EFP, Midcycle, or MLP) were evaluated through multiple comparisons using Steel–Dwass test. The values are expressed as mean (SD).

*Abbreviations*: *3βAdiol*, 5α-androstane-3β,17β-diol; *Δ5-diol*, androstenediol; *DHEA*, dehydroepiandrosterone; *E2*, 17β-estradiol; *EFP*, early follicular phase; *Midcycle*, mid-cycle phase; *MLP*, mid-luteal phase

### Sex differences in mood (Table 4)

Women in the EFP had significantly higher mean scores on HAM-D than in men (p = 0.0197). Statistically significant differences in scores on HAM-D were not observed between men and women in the Midcycle (p = 0.1708) or between men and women in the MLP (p = 0.1381). The scores for each subitem of HAM-D, BDI-II, and QIDS-J are shown in S2 Table.

**Table 4:** Sex differences in scores on depression rating scales.

	Men (n = 25)	Women			*Kruskal–Wallis test*	*Steel–Dwass test*
		EFP (n = 23)	Midcycle (n = 22)	MLP (n = 22)		
HAM-D	1.1 (1.7)	3.0 (2.6)	1.4 (1.7)	1.9 (2.5)	**p = 0.0162**	**Men vs EFP (p = 0.0197)**
BDI-II	3.2 (5.4)	5.9 (6.1)	3.0 (3.7)	3.4 (3.8)	p = 0.1708	
QIDS-J	1.68 (2.3)	3.5 (3.3)	2.3 (2.6)	2.3 (2.2)	p = 0.1381	

Scores on three depression rating scales (HAM-D, BDI-II, and QIDS-J) were compared between men and women in the EFP, Midcycle, and MLP. Four group mean comparisons were performed using Kruskal–Wallis test. If the results of Kruskal–Wallis test were significant, differences between pairs of men and women (in one of EFP, Midcycle, or MLP) were evaluated through multiple comparisons using Steel–Dwass test. The values are expressed as mean (SD).

*Abbreviations*: *BDI-II*, Beck Depression Inventory-II; *EFP*, early follicular phase; *HAM-D*, Hamilton Rating Scale for Depression 21 items; *Midcycle*, mid-cycle phase; *MLP*, mid-luteal phase; *QIDS-J*, Quick Inventory of Depressive Symptomatology-Japanese version

### Comparison of steroid levels between the young and the elderly (Tables 5 and 6)

The steroid levels of the young population in this study were compared to those of the geriatric population in our previously published study [8]. The serum levels of 3βAdiol, Δ5-diol, DHEA, and E2 in men and women (in the EFP, Midcycle, and MLP) were higher in the young population than in the geriatric population. Serum 3βAdiol level was one and half times higher in men and two times higher in women in the young population than in the geriatric population. Further, serum Δ5-diol level was two times higher in men and two and half times higher in women in the young population than in the geriatric population.

**Table 5:** Comparison of steroid levels between young men and elderly men.

	young men (n = 25)	elderly men (n = 12)	*Mann–Whitney test*
3βAdiol (pg/mL)	38.02 (11.13)	25.08 (20.06)	**p = 0.0021**
Δ5-diol (ng/mL)	1.04 (0.36)	0.50 (0.31)	**p = 0.0002**
DHEA (ng/mL)	4.70 (2.40)	1.87 (0.65)	**p<0.0001**
E2 (pg/mL)	21.00 (4.71)	15.87 (5.56)	**p = 0.0055**

Serum levels of four steroids (3βAdiol, Δ5-diol, DHEA, and E2) were compared between young men and elderly men using Mann–Whitney test. Data on the elderly population were from our previous publication [8]. The values are expressed as mean (SD).

*Abbreviations*: *3βAdiol*, 5α-androstane-3β,17β-diol; *Δ5-diol*, androstenediol; *DHEA*, dehydroepiandrosterone; *E2*, 17β-estradiol

**Table 6:** Comparison of steroid levels between young women and elderly women.

	young women			elderly women (n = 16)	*Kruskal–Wallis test*	*Steel–Dwass test*
	EFP (n = 23)	Midcycle (n = 22)	MLP (n = 22)			
3βAdiol (pg/mL)	10.22 (3.72)	12.80 (7.31)	11.85 (4.60)	5.69 (7.60)	**p<0.0001**	**elderly vs EFP (p = 0.0018)**
						**elderly vs Midcycle (p = 0.0006)**
						**elderly vs MLP (p = 0.0007)**
Δ5-diol (ng/mL)	0.58 (0.20)	0.62 (0.40)	0.52 (0.18)	0.23 (0.07)	**p<0.0001**	**elderly vs EFP (p<0.0001)**
						**elderly vs Midcycle (p<0.0001)**
						**elderly vs MLP (p<0.0001)**
DHEA (ng/mL)	5.06 (2.35)	4.36 (1.49)	4.43 (2.33)	2.24 (0.83)	**p<0.0001**	**elderly vs EFP (p<0.0001)**
						**elderly vs Midcycle (p = 0.0001)**
						**elderly vs MLP (p = 0.0008)**
E2 (pg/mL)	40.14 (41.81)	187.91 (135.78)	163.86 (149.25)	2.24 (1.39)	**p<0.0001**	**elderly vs EFP (p<0.0001)**
						**elderly vs Midcycle (p<0.0001)**
						**elderly vs MLP (p<0.0001)**

Serum levels of four steroids (3βAdiol, Δ5-diol, DHEA, and E2) were compared between elderly women and young women (in the EFP, Midcycle, and MLP) using Kruskal–Wallis test followed by Steel–Dwass test. Data on the elderly population were from our previous publication [8]. The values are expressed as mean (SD).

*Abbreviations*: *3βAdiol*, 5α-androstane-3β, 17β-diol; *Δ5-diol*, androstenediol; *DHEA*, dehydroepiandrosterone; *E2*, 17β-estradiol; *EFP*, early follicular phase; *Midcycle*, mid-cycle phase; *MLP*, mid-luteal phase

### Association between steroid levels and mood (S1 Fig)

In men, a correlation was found between serum levels of 3βAdiol and E2 (ρ = 0.5005, p = 0.0108; S1A Fig). In addition, a correlation was found between the serum levels of 3βAdiol and Δ5-diol only in young women in the Midcycle (ρ = 0.4648, p = 0.0293; S1C Fig). Furthermore, a significant correlation was found between the serum levels of Δ5-diol and DHEA in both sexes. Except for a correlation between serum Δ5-diol level and score on QIDS-J in men (ρ = 0.4041, p = 0.0451), no other significant correlations were observed between serum steroid levels and scores on depression inventories. Strong correlations in scores were observed between the three depression rating scales.

## Discussion

To the best of our knowledge, this is the first study to present comprehensive data on serum levels of 3βAdiol and Δ5-diol in humans. The sensitivity and specificity of assays are particularly important when measuring steroid hormone levels that are unknown or are expected to be low. Any concerns regarding the sensitivity and specificity of assays were overcome by utilizing the well-validated LC–MS/MS system. However, the high cost of the LC–MS/MS assay and the limited number of facilities where this assay can be performed are issues that need to be addressed in the future. Another concern is the difficulty associated with studying women of reproductive age with regard to their menstrual cycles. However, careful menstrual recording and use of LH surge detection kits allowed for successful and accurate blood sampling at appropriate time-points. In women, E2 levels changed dynamically, while DHEA levels remained constant and unchanged throughout the menstrual cycle. E2 levels were lower in men than in women, and there were no significant differences in DHEA levels between the sexes. These results are consistent with those of previous studies [13–15].

In women, serum 3βAdiol level fluctuated slightly, and its amplitude of change was smaller than that of serum E2 level. Serum Δ5-diol levels were constant throughout menstruation. Further, the plot of the scores of women on the depression rating scales yielded a V-shaped curve, as the lowest and highest scores were obtained during ovulation and menstruation, respectively. If we assume that ERβ agonists influence the mood of women during their menstrual cycle, then the main factors are likely to be serum 3βAdiol and E2 levels, not serum Δ5-diol level. In this study, the extent of the effect of serum 3βAdiol level on the mood of women during their menstrual cycle is not covered. To determine the extent of the effects of 3βAdiol level and serum E2 level on the mood of women, it is necessary to conduct a larger study that includes patients with pre-menstrual syndrome and patients with pre-menstrual dysphoric disorder.

Men had higher serum levels of 3βAdiol and Δ5-diol than women. The HAM-D scores of women in the EFP were significantly higher than those of men, and this trend is comparable to those of BDI-II scores and QIDS-J scores. If the low HAM-D scores of men are due to ERβ agonists, then the protective effect of ERβ agonists reflected by the low HAM-D scores can be attributed to serum 3βAdiol and Δ5-diol levels, not serum E2 levels.

One of the major questions regarding depression is the sex difference in its prevalence [20]. Women were almost twice as likely as men to have depression [21]. However, neuroendocrinological explanations for this question are still controversial. Recent studies have reported no relationship between testosterone levels and depression [22–24]. Therefore, it is difficult to explain the sex differences in the prevalence of depression based on testosterone levels alone; this suggests that yet-unknown factors may protect men from depression. Based on our findings in this study, we hypothesize that serum 3βAdiol and Δ5-diol levels are one of the protective factors that protect men from depression. In women, on the other hand, E2 is the central sex hormone that supports their mood, and it is not compensated by low levels of 3βAdiol and Δ5-diol. Hence, during menstruation (i.e., when E2 levels are at their lowest), women tend to be depressed.

In this study, a direct negative correlation was not observed between the serum levels of these steroids and the scores on depression rating scales, and this may be due to two reasons. First, this study included only healthy subjects, which may have resulted in a narrow range of scores on the depression rating scales. Although there are ethical issues to consider, if patients with depression were included in this study, we may have found negative correlations. Second, it is possible that 3βAdiol and Δ5-diol are not state markers of depression severity, but trait markers of depression vulnerability. It is therefore necessary to conduct a prospective study in which the serum 3βAdiol and Δ5-diol levels of healthy subjects are measured, and the participants are followed up. Serum 3βAdiol and Δ5-diol levels can then be compared between subjects who developed depression and subjects who did not develop depression. Further research may also clarify the significance of the difference in serum 3βAdiol and Δ5-diol levels between the young and the elderly.

The present study has several limitations that should be acknowledged. First, the sample size was small, and most of the subjects were medical students and staff at the university hospital, which hinders the generalization of the study findings to the general population. In addition, we did not measure other important hormones that affect mood in women, such as progesterone. Despite these limitations, the large sex differences in serum levels of 3βAdiol and Δ5-diol observed in the present study are important findings for furthering our understanding of the pathophysiology of depression, which may lead to the development of new treatments and preventative approaches for depression.

## Supporting information

S1 Fig Spearman’s correlation coefficients between serum steroid levels and scores on depression inventories. Panel A: Men. B: Women in the EFP. C: Women in the Midcycle. D: Women in the MLP. The distribution of each variable is shown on the diagonal. Below the diagonal, the scatterplot matrixes are displayed. Above the diagonal, the values of the Spearman’s correlation coefficients (ρ) and p values are shown. *Abbreviations*: *3βAdiol*, 5α-androstane-3β,17β-diol; *BDI-II*, Beck Depression Inventory-II; *Δ5-diol*, androstenediol; *DHEA*, dehydroepiandrosterone; *E2*, 17β-estradiol; *EFP*, early follicular phase; *HAM-D*, Hamilton Rating Scale for Depression 21 items; *Midcycle*, mid-cycle phase; *MLP*, mid-luteal phase; *QIDS-J*, Quick Inventory of Depressive Symptomatology-Japanese version.
(PDF)

S1 Table Scores for each subitem of the depression rating scales during the menstrual cycle. The differences at the three time-points in the menstrual cycle (EFP, Midcycle, and MLP) were assessed using the Friedman test. Post hoc analysis was conducted using Wilcoxon signed-rank test, with application of the Bonferroni correction. The values are expressed as mean (SD). *Abbreviations*: *BDI-II*, Beck Depression Inventory-II; *EFP*, early follicular phase; *HAM-D*, Hamilton Rating Scale for Depression 21 items; *Midcycle*, mid-cycle phase; *MLP*, mid-luteal phase; *QIDS-J*, Quick Inventory of Depressive Symptomatology-Japanese version; *NA*, not applicable.
(PDF)

S2 Table Sex differences in scores for each subitem of the depression rating scales. The scores on three depression rating scales (HAM-D, BDI-II, and QIDS-J) were compared between men and women in the EFP, Midcycle, and MLP. Four group mean comparisons were performed using Kruskal–Wallis test. If the results of Kruskal–Wallis test were significant, differences between pairs of men and women (in one of EFP, Midcycle, or MLP) were evaluated through multiple comparisons using Steel–Dwass test. The values are expressed as mean (SD). *Abbreviations*: *BDI-II*, Beck Depression Inventory-II; *EFP*, early follicular phase; *HAM-D*, Hamilton Rating Scale for Depression 21 items; *Midcycle*, mid-cycle phase; *MLP*, mid-luteal phase; *QIDS-J*, Quick Inventory of Depressive Symptomatology-Japanese version.
(PDF)
