# Supplementary material for: Sex differences in serum levels of 5α-androstane-3β, 17β-diol, and androstenediol in the young adults: A liquid chromatography–tandem mass spectrometry study
Source: PLoS One. 2021 Dec 15;16(12):e0261440. doi: 10.1371/journal.pone.0261440 (PMC8673626; doi:10.1371/journal.pone.0261440)
Supplement: S1 Fig — Panel A: Men. B: Women in the EFP. C: Women in the Midcycle. D: Women in the MLP. The distribution of each variable is shown on the diagonal. Below the diagonal, the scatterplot matrixes are displayed. Above the diagonal, the values of the Spearman’s correlation coefficients (ρ) and p values are shown. Abbreviations: 3βAdiol, 5α-androstane-3β,17β-diol; BDI-II, Beck Depression Inventory-II; Δ5-diol, androstenediol; DHEA, dehydroepiandrosterone; E2, 17β-estradiol; EFP, early follicular phase; HAM-D, Hamilton Rating Scale for Depression 21 items; Midcycle, mid-cycle phase; MLP, mid-luteal phase; QIDS-J, Quick Inventory of Depressive Symptomatology-Japanese version. (PDF) [file pone.0261440.s001.pdf]

S1 Fig. Spearman’ s correlation coefficients between serum steroid levels and scores on depression inventories

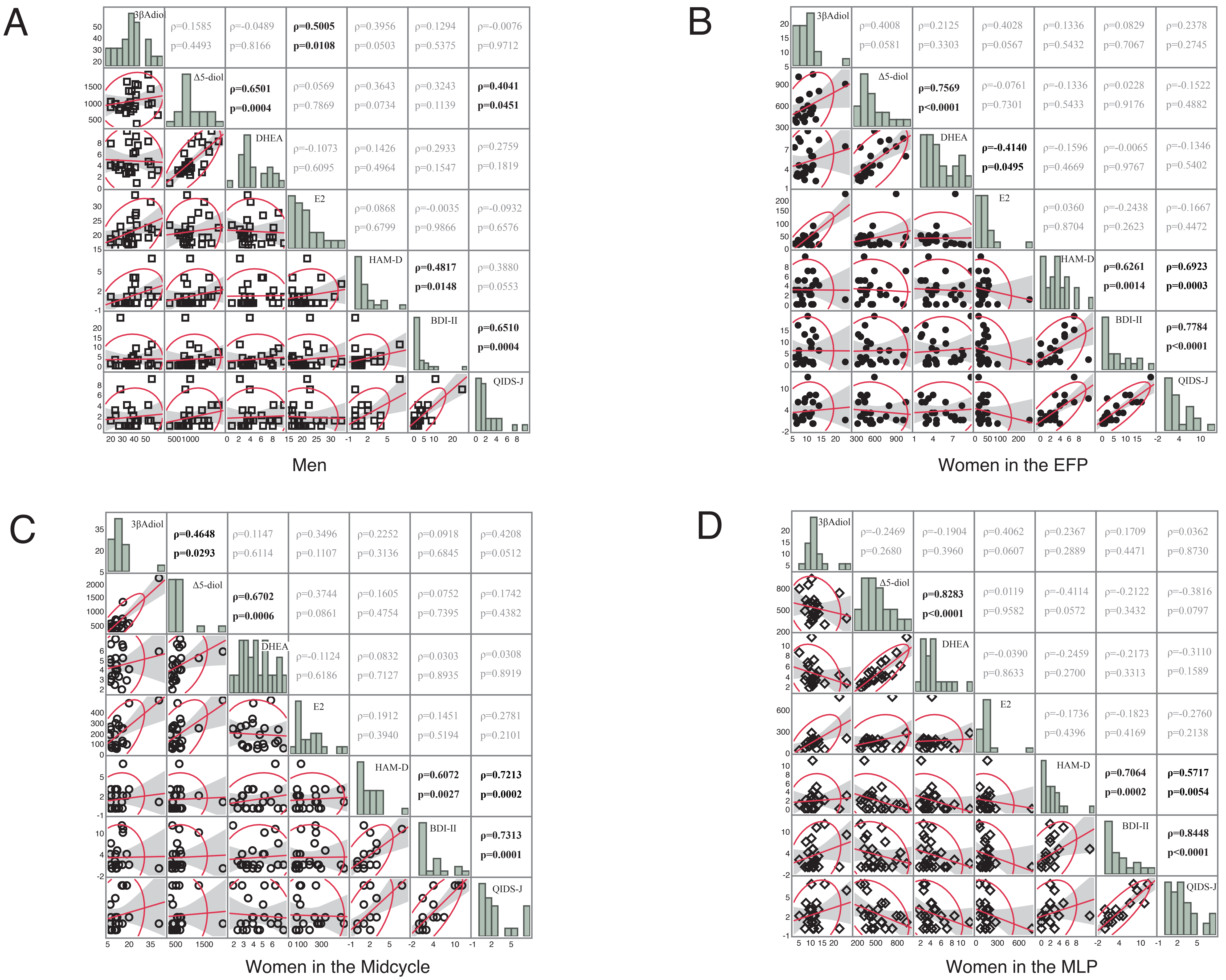

Panel A: Men. B: Women in the EFP. C: Women in the Midcycle. D: Women in the MLP.

The distribution of each variable is shown on the diagonal. Below the diagonal, the scatterplot matrixes are displayed. Above the diagonal, the values of the Spearman’ s correlation coefficients (ρ) and p values are shown. *Abbreviations: 3βAdiol,* 5α-androstane-3β,17β-diol; *BDI-II,* Beck Depression Inventory-II; *Δ5-diol,* androstenediol; *DHEA,* dehydroepiandrosterone; *E2,* 17βestradiol; *EFP,* early follicular phase; *HAM-D,* Hamilton Rating Scale for Depression 21 items; Midcycle, mid-cycle phase; *MLP,* mid-luteal phase; *QIDS-J,* Quick Inventory of Depressive Symptomatology-Japanese version
