# Supplementary material for: Sex differences in serum levels of 5α-androstane-3β, 17β-diol, and androstenediol in the young adults: A liquid chromatography–tandem mass spectrometry study
Source: PLoS One. 2021 Dec 15;16(12):e0261440. doi: 10.1371/journal.pone.0261440 (PMC8673626; doi:10.1371/journal.pone.0261440)
Supplement: S1 Table — The differences at the three time-points in the menstrual cycle (EFP, Midcycle, and MLP) were assessed using the Friedman test. Post hoc analysis was conducted using Wilcoxon signed-rank test, with application of the Bonferroni correction. The values are expressed as mean (SD). Abbreviations: BDI-II, Beck Depression Inventory-II; EFP, early follicular phase; HAM-D, Hamilton Rating Scale for Depression 21 items; Midcycle, mid-cycle phase; MLP, mid-luteal phase; QIDS-J, Quick Inventory of Depressive Symptomatology-Japanese version; NA, not applicable. (PDF) [file pone.0261440.s002.pdf]

S1 Table. Scores for each subitem of the depression rating scales during the menstrual cycle

HAM-D

	Women			*Friedman test*	*Wilcoxon signed-rank test (with Bonferroni correction)*
	EFP (n=22)	Midcycle (n=22)	MLP (n=22)		
**HAM-D**	**3.2 (2.6)**	**1.4 (1.7)**	**1.9 (2.5)**	**p=0.0066**	**EFP vs Midcycle (p=0.012)**<br>**EFP vs MLP (p=0.045)**
1. Depressed mood	0.4 (0.5)	0.2 (0.6)	0.2 (0.5)	p=0.1482	
**2. Feelings of guilt**	**0.4 (0.5)**	**0.0 (0.2)**	**0.2 (0.4)**	**p=0.0051**	**EFP vs Midcycle (p=0.032)**
3. Suicide	0.0 (0.2)	0 (0)	0.1 (0.4)	p=0.6065	
4. Insomnia - Initial	0.3 (0.5)	0.1 (0.4)	0.1 (0.4)	p=0.3247	
5. Insomnia - Middle	0.1 (0.3)	0.1 (0.3)	0.0 (0.2)	p=0.7788	
**6. Insomnia - Delayed**	**0.2 (0.4)**	**0.0 (0.2)**	**0 (0)**	**p=0.0388**	
7.Work and interests	0.3 (0.5)	0.1 (0.3)	0.1 (0.5)	p=0.1017	
8. Retardation	0 (0)	0 (0)	0 (0)	NA	
9. Agitation	0 (0)	0 (0)	0 (0)	NA	
10. Anxiety - Psychic	0.4 (0.6)	0.1 (0.4)	0.3 (0.6)	p=0.1496	
11. Anxiety - Somatic	0.4 (0.6)	0.2 (0.4)	0.3 (0.5)	p=0.7408	
12. Somatic symptoms - Gastrointestinal	0 (0)	0.0 (0.2)	0 (0)	p=0.3679	
13. Somatic symptoms - General	0.4 (0.6)	0.2 (0.4)	0.3 (0.5)	p=0.6065	
14. Genital symptoms	0.1 (0.3)	0.0 (0.2)	0 (0)	p=0.2231	
15. Hypochondriasis	0.0 (0.2)	0 (0)	0 (0)	p=0.3679	
16. Weight loss	0 (0)	0 (0)	0 (0)	NA	
17. Insight	0 (0)	0 (0)	0 (0)	NA	
18. Diurnal variation	0.2 (0.4)	0.0 (0.2)	0.1 (0.3)	p=0.3114	
19. Depersonalization and derealization	0 (0)	0 (0)	0 (0)	NA	
20. Paranoid symptoms	0 (0)	0 (0)	0 (0)	NA	
21. Obsessional symptoms	0.0 (0.2)	0.0 (0.2)	0.0 (0.2)	NA	

BDI-II

	Women			Friedman test	Wilcoxon signed-rank test (with Bonferroni correction)
	EFP (n=22)	Midcycle (n=22)	MLP (n=22)		
**BDI-II**	**6.2 (6.1)**	**3.0 (3.7)**	**3.4 (3.8)**	**p=0.0011**	**EFP vs Midcycle (p=0.0067)**
1. Sadness	0.5 (0.6)	0.4 (0.5)	0.3 (0.5)	p=0.0672	
2. Pessimism	0.3 (0.5)	0.2 (0.4)	0.1 (0.4)	p=0.1561	
3. Past failure	0.2 (0.4)	0.2 (0.4)	0.2 (0.4)	p=0.8669	
4. Loss of pleasure	0.2 (0.4)	0.1 (0.3)	0.1 (0.3)	p=0.8465	
5. Guilty feelings	0.2 (0.4)	0.1 (0.4)	0.1 (0.3)	p=0.2466	
6. Punishment feelings	0 (0)	0 (0)	0 (0)	NA	
7. Self-dislike	0.4 (0.7)	0.2 (0.4)	0.2 (0.4)	p=0.4216	
8. Self-criticalness	0.3 (0.6)	0.2 (0.4)	0.1 (0.3)	p=0.2504	
9. Suicidal thoughts or wishes	0.0 (0.2)	0 (0)	0 (0)	p=0.3679	
**10. Crying**	**0.4 (0.6)**	**0.1 (0.4)**	**0 (0)**	**p=0.0022**	**EFP vs MLP (p=0.025)**
11. Agitation	0.2 (0.4)	0.0 (0.2)	0.1 (0.3)	p=0.3679	
12. Loss of interest	0.2 (0.4)	0.1 (0.4)	0.0 (0.2)	p=0.1353	
**13. Indecisiveness**	**0.3 (0.6)**	**0.1 (0.3)**	**0 (0)**	**p=0.0061**	
14. Worthlessness	0.2 (0.4)	0.1 (0.4)	0.0 (0.2)	p=0.3114	
**15. Loss of energy**	**0.5 (0.5)**	**0.1 (0.4)**	**0.3 (0.5)**	**p=0.0346**	**EFP vs Midcycle (p=0.032)**
16. Change in sleeping pattern	0.5 (0.7)	0.5 (0.5)	0.4 (0.7)	p=0.7788	
**17. Irritability**	**0.5 (0.6)**	**0.1 (0.4)**	**0.3 (0.5)**	**p=0.0275**	**EFP vs Midcycle (p=0.04)**
**18. Change in appetite**	**0.3 (0.5)**	**0.1 (0.3)**	**0.5 (0.6)**	**p=0.0036**	**Midcycle vs MLP (p=0.044)**
**19. Concentration difficulty**	**0.4 (0.5)**	**0.1 (0.3)**	**0.1 (0.4)**	**p=0.0451**	
20. Tiredness or fatigue	0.5 (0.5)	0.2 (0.4)	0.5 (0.6)	p=0.0627	
21. Loss of interest in sex	0.1 (0.4)	0.0 (0.2)	0 (0)	p=0.0970	

QIDS-J

	Women			Friedman test	Wilcoxon signed-rank test (with Bonferroni correction)
	EFP (n=22)	Midcycle (n=22)	MLP (n=22)		
**QIDS-J**	**3.6 (3.3)**	**2.3 (2.6)**	**2.3 (2.2)**	**p=0.017**	
1. Sleep items	0.9 (1.0)	0.7 (0.9)	0.6 (1.0)	p=0.4692	
2. Mood (Sad)	0.2 (0.4)	0.2 (0.4)	0.2 (0.4)	p=0.8669	
3. Appetite/Weight change items	0.6 (0.8)	0.5 (0.7)	0.6 (0.8)	p=0.5188	
4. Concentration/Decision making	0.5 (0.6)	0.2 (0.5)	0.2 (0.4)	p=0.0627	
5. Outlook (Self)	0.4 (0.7)	0.2 (0.4)	0.1 (0.3)	p=0.1778	
6. Suicidal Ideation	0.1 (0.5)	0.1 (0.3)	0 (0)	p=0.2725	
7. Involvement	0.2 (0.4)	0.1 (0.3)	0.1 (0.3)	p=0.1054	
**8. Energy/Fatigability**	**0.5 (0.6)**	**0.1 (0.4)**	**0.5 (0.6)**	**p=0.0165**	**EFP vs Midcycle (p=0.044)**
9. Psychomotor items	0.1 (0.3)	0.1 (0.3)	0.1 (0.3)	p=1.0000	

The differences at the three time-points in the menstrual cycle (EFP, Midcycle, and MLP) were assessed using the Friedman test. Post hoc analysis was conducted using Wilcoxon signed-rank test, with application of the Bonferroni correction. The values are expressed as mean (SD).

Abbreviations: BDI-II, Beck Depression Inventory-II; *EFP,* early follicular phase; *HAM-D,* Hamilton Rating Scale for Depression 21 items; *Midcycle,* mid-cycle phase; *MLP,* mid-luteal phase; *QIDS-J,* Quick Inventory of Depressive Symptomatology-Japanese version; *NA,* not applicable
