# Supplementary material for: Sex differences in serum levels of 5α-androstane-3β, 17β-diol, and androstenediol in the young adults: A liquid chromatography–tandem mass spectrometry study
Source: PLoS One. 2021 Dec 15;16(12):e0261440. doi: 10.1371/journal.pone.0261440 (PMC8673626; doi:10.1371/journal.pone.0261440)
Supplement: S2 Table — The scores on three depression rating scales (HAM-D, BDI-II, and QIDS-J) were compared between men and women in the EFP, Midcycle, and MLP. Four group mean comparisons were performed using Kruskal–Wallis test. If the results of Kruskal–Wallis test were significant, differences between pairs of men and women (in one of EFP, Midcycle, or MLP) were evaluated through multiple comparisons using Steel–Dwass test. The values are expressed as mean (SD). Abbreviations: BDI-II, Beck Depression Inventory-II; EFP, early follicular phase; HAM-D, Hamilton Rating Scale for Depression 21 items; Midcycle, mid-cycle phase; MLP, mid-luteal phase; QIDS-J, Quick Inventory of Depressive Symptomatology-Japanese version. (PDF) [file pone.0261440.s003.pdf]

S2 Table. Sex differences in scores for each subitem of the depression rating scales

HAM-D

	Men (n=25)	Women			*Kruskal–Wallis test*	*Steel–Dwass test*
		EFP (n=23)	Midcycle (n=22)	MLP (n=22)		
**HAM-D**	**1.1 (1.7)**	**3.0 (2.6)**	**1.4 (1.7)**	**1.9 (2.5)**	**p=0.0162**	**Men vs EFP (p=0.0197)**
1. Depressed mood	0.1 (0.3)	0.4 (0.5)	0.2 (0.6)	0.2 (0.5)	p=0.1231	
**2. Feelings of guilt**	**0.0 (0.2)**	**0.3 (0.5)**	**0.0 (0.2)**	**0.2 (0.4)**	**p=0.0105**	**Men vs EFP (p=0.0364)**
3. Suicide	0 (0)	0.0 (0.2)	0 (0)	0.1 (0.4)	p=0.5486	
4. Insomnia - Initial	0.0 (0.2)	0.3 (0.4)	0.1 (0.4)	0.1 (0.4)	p=0.1886	
5. Insomnia - Middle	0.1 (0.3)	0.1 (0.3)	0.1 (0.3)	0.0 (0.2)	p=0.9398	
6. Insomnia - Delayed	0.2 (0.4)	0.2 (0.4)	0.0 (0.2)	0 (0)	p=0.1321	
7.Work and interests	0.1 (0.3)	0.3 (0.4)	0.1 (0.3)	0.1 (0.5)	p=0.3222	
8. Retardation	0 (0)	0 (0)	0 (0)	0 (0)	p=1.0000	
9. Agitation	0.1 (0.4)	0 (0)	0 (0)	0 (0)	p=0.4436	
10. Anxiety - Psychic	0.2 (0.4)	0.4 (0.6)	0.1 (0.4)	0.3 (0.6)	p=0.3468	
11. Anxiety - Somatic	0.1 (0.2)	0.3 (0.6)	0.2 (0.4)	0.3 (0.5)	p=0.1000	
12. Somatic symptoms - Gastrointestinal	0 (0)	0 (0)	0.0 (0.2)	0 (0)	p=0.3644	
13. Somatic symptoms - General	0.2 (0.4)	0.4 (0.6)	0.2 (0.4)	0.3 (0.5)	p=0.4124	
14. Genital symptoms	0.0 (0.2)	0.1 (0.3)	0.0 (0.2)	0 (0)	p=0.5657	
15. Hypochondriasis	0 (0)	0.0 (0.2)	0 (0)	0 (0)	p=0.3916	
16. Weight loss	0 (0)	0 (0)	0 (0)	0 (0)	p=1.0000	
17. Insight	0 (0)	0 (0)	0 (0)	0 (0)	p=1.0000	
18. Diurnal variation	0.0 (0.2)	0.2 (0.4)	0.0 (0.2)	0.1 (0.3)	p=0.3435	
19. Depersonalization and derealization	0 (0)	0 (0)	0 (0)	0 (0)	p=1.0000	
20. Paranoid symptoms	0 (0)	0 (0)	0 (0)	0 (0)	p=1.0000	
21. Obsessional symptoms	0 (0)	0.0 (0.2)	0.0 (0.2)	0.0 (0.2)	p=0.7659	

BDI-II

	Men (n=25)	Women			*Kruskal–Wallis test*	*Steel–Dwass test*
		EFP (n=23)	Midcycle (n=22)	MLP (n=22)		
BDI-II	3.2 (5.4)	5.9 (6.1)	3.0 (3.7)	3.4 (3.8)	p=0.1708	
1. Sadness	0.3 (0.5)	0.5 (0.6)	0.4 (0.5)	0.3 (0.5)	p=0.3453	
2. Pessimism	0.2 (0.5)	0.3 (0.5)	0.2 (0.4)	0.1 (0.4)	p=0.3973	
3. Past failure	0.3 (0.7)	0.2 (0.4)	0.2 (0.4)	0.2 (0.4)	p=0.9114	
4. Loss of pleasure	0.1 (0.3)	0.1 (0.3)	0.1 (0.3)	0.1 (0.3)	p=0.9430	
5. Guilty feelings	0.2 (0.5)	0.2 (0.4)	0.1 (0.4)	0.1 (0.3)	p=0.6694	
6. Punishment feelings	0.1 (0.4)	0 (0)	0 (0)	0 (0)	p=0.1436	
7. Self-dislike	0.3 (0.7)	0.4 (0.7)	0.2 (0.4)	0.2 (0.4)	p=0.8512	
8. Self-criticalness	0.2 (0.5)	0.3 (0.6)	0.2 (0.4)	0.1 (0.3)	p=0.6036	
9. Suicidal thoughts or wishes	0.0 (0.2)	0.0 (0.2)	0 (0)	0 (0)	p=0.6019	
10. Crying	0.1 (0.3)	0.4 (0.6)	0.1 (0.4)	0 (0)	p=0.0112	
11. Agitation	0 (0)	0.2 (0.4)	0.0 (0.2)	0.1 (0.3)	p=0.1391	
12. Loss of interest	0.1 (0.3)	0.2 (0.4)	0.1 (0.4)	0.0 (0.2)	p=0.3043	
13. Indecisiveness	0.1 (0.3)	0.3 (0.6)	0.1 (0.3)	0 (0)	p=0.0364	
14. Worthlessness	0.1 (0.6)	0.2 (0.4)	0.1 (0.4)	0.0 (0.2)	p=0.3517	
15. Loss of energy	0.2 (0.4)	0.4 (0.5)	0.1 (0.4)	0.3 (0.5)	p=0.1579	
16. Change in sleeping pattern	0.4 (0.5)	0.5 (0.7)	0.5 (0.5)	0.4 (0.7)	p=0.9322	
**17. Irritability**	**0 (0)**	**0.5 (0.6)**	**0.1 (0.4)**	**0.3 (0.5)**	**p=0.0014**	**Men vs EFP (p=0.0015)**<br>**Men vs MLP (p=0.0140)**
**18. Change in appetite**	**0.1 (0.3)**	**0.3 (0.5)**	**0.1 (0.3)**	**0.5 (0.6)**	**p=0.0133**	**Men vs MLP (p=0.0416)**
19. Concentration difficulty	0.1 (0.3)	0.3 (0.5)	0.1 (0.3)	0.1 (0.4)	p=0.0878	
20. Tiredness or fatigue	0.3 (0.5)	0.4 (0.5)	0.2 (0.4)	0.5 (0.6)	p=0.1542	
21. Loss of interest in sex	0.0 (0.2)	0.1 (0.3)	0.0 (0.2)	0 (0)	p=0.2674	

QIDS-J

	Men (n=25)	Women			*Kruskal–Wallis test*	*Steel–Dwass test*
		EFP (n=23)	Midcycle (n=22)	MLP (n=22)		
QIDS-J	1.68 (2.3)	3.5 (3.3)	2.3 (2.6)	2.3 (2.2)	p=0.1381	
1. Sleep items	0.6 (0.7)	0.8 (1.0)	0.7 (0.9)	0.6 (1.0)	p=0.8978	
2. Mood (Sad)	0.1 (0.3)	0.2 (0.4)	0.2 (0.4)	0.2 (0.4)	p=0.5206	
3. Appetite/Weight change items	0.2 (0.5)	0.6 (0.8)	0.5 (0.7)	0.6 (0.8)	p=0.1546	
4. Concentration/Decision making	0.2 (0.4)	0.5 (0.6)	0.2 (0.5)	0.2 (0.4)	p=0.1684	
5. Outlook (Self)	0.1 (0.4)	0.4 (0.7)	0.2 (0.4)	0.1 (0.3)	p=0.0630	
6. Suicidal Ideation	0.0 (0.2)	0.1 (0.5)	0.1 (0.3)	0 (0)	p=0.4931	
7. Involvement	0.2 (0.5)	0.2 (0.4)	0.1 (0.3)	0.1 (0.3)	p=0.5586	
8. Energy/Fatigability	0.2 (0.4)	0.5 (0.6)	0.1 (0.4)	0.5 (0.6)	p=0.0479	
9. Psychomotor items	0.0 (0.2)	0.1 (0.3)	0.1 (0.3)	0.1 (0.3)	p=0.8890	

The scores on three depression rating scales (HAM-D, BDI-II, and QIDS-J) were compared between men and women in the EFP, Midcycle, and MLP. Four group mean comparisons were performed using Kruskal–Wallis test. If the results of Kruskal–Wallis test were significant, differences between pairs of men and women (in one of EFP, Midcycle, or MLP) were evaluated through multiple comparisons using Steel–Dwass test. The values are expressed as mean (SD).

Abbreviations: BDI-II, Beck Depression Inventory-II; *EFP,* early follicular phase; *HAM-D,* Hamilton Rating Scale for Depression 21 items; *Midcycle,* mid-cycle phase; *MLP,* mid-luteal phase; *QIDS-J,* Quick Inventory of Depressive Symptomatology-Japanese version
